# Vitamin E succinate and cancer treatment: a vitamin E prototype for selective antitumour activity

**DOI:** 10.1038/sj.bjc.6601360

**Published:** 2003-11-11

**Authors:** J Neuzil

**Affiliations:** 1 School of Health Sciences, Griffith University, Southport 9726, Queensland, Australia; 2 Department of Pathology II, University Hospital, Linkoping, Sweden

**Keywords:** *α*-tocopheryl succinate, vitamin E analogues, apoptosis, neoplastic disease, pharmacokinetics

## Abstract

Great hope has been given to micronutrients as anticancer agents, since they present natural compounds with beneficial effects for normal cells and tissues. One of these is vitamin E (VE), an antioxidant and an essential component of biological membranes and circulating lipoproteins. In spite of a number of epidemiological and intervention studies, little or no correlation between VE intake and incidence of cancer has been found. Recent reports have identified a redox-silent analogue of VE, *α*-tocopheryl succinate (*α*-TOS), as a potent anticancer agent with a unique structure and pharmacokinetics *in vivo*. *α*-TOS is highly selective for malignant cells, inducing them into apoptotic death largely via the mitochondrial route. The molecule of *α*-TOS may be modified so that analogues with higher activity are generated. Finally, *α*-TOS and similar agents are metabolised to VE, thereby yielding a compound with a secondary beneficial activity. Thus, *α*-TOS epitomises a group of novel compounds that hold substantial promise as future anticancer drugs. The reasons for this optimistic notion are discussed in the following paragraphs.

## VITAMIN E (VE) IS A POOR ANTICANCER AGENT

Numerous epidemiological and intervention studies tested the possibility that VE suppresses cancer (see e.g. Albanes *et al*, 1996,^2000^; Heinonen *et al*, 1998; Heart Protection Study Collaborative Group, 2002). The idea has been attractive, as VE is a micronutrient with high antioxidant activity. To a certain degree, the level of VE can be manipulated by dietary supplementation. As the hepatic system prevents both VE hyper- and hypovitaminosis, maintaining the circulating/tissue levels of VE within a narrow margin, pathologies associated with too much or too little VE, are rare. Therefore, it is apparent that diseases linked to VE deficiency will only occur in association with infrequent syndromes of impairment of VE intake and its maintenance, such as abetalipoproteinaemia or the familial isolated VE deficiency syndrome. On the other hand, given the relatively narrow margin between minimal and maximal VE levels in circulating lipoproteins and in tissues, there is only a limited scope for VE manipulation by intervention therapy. Hence, it is not surprising that little or no association between disease incidence, including neoplasia, and VE intake has been repeatedly observed (Albanes *et al*, 1996,^2000^; Heart Protection Study Collaborative Group, 2002). Attempts to find a correlation between VE supplementation and pathology may have failed as, following VE ingestion, its level raises only transiently, the ‘excess’ VE being efficiently removed from the body by the hepatic system armed with the saturable *α*-tocopherol (*α-*TOH) transfer protein (Brigelius-Flohe *et al*, 2002).

## *α*-TOCOPHERYL SUCCINATE (*α*-TOS) IS AN ANTICANCER VE ANALOGUE DUE TO ITS APOPTOGENIC ACTIVITY

Probably, the main reason why VE as such exerts little or no anticancer effect may be due to its biological activity, since it is a redox-active substance, which does not cause, unlike many anticancer agents, apoptosis of malignant cells (Neuzil *et al*, 1999). This premise, however, has been found for semisynthetic analogues of VE, as documented in a number of recent reports for *α*-TOS (Fariss *et al*, 1994; Neuzil *et al*, 1999; Pussinen *et al*, 2000; Neuzil *et al*, 2001a,2001b,2001c; Yu *et al*, 2001). The mechanism by which *α*-TOS kills cancer cells is not completely understood. It has been shown that this occurs by inducing apoptosis in malignant cells primarily by mitochondrial destabilisation (Alleva *et al*, 2001; Kogure *et al*, 2001; Neuzil *et al*, 2001c; Weber *et al*, 2003), a process amplified by the modulation of signalling pathways (Qian *et al*, 1997; Neuzil *et al*, 2001c; Yu *et al*, 2001). Mitochondrial destabilisation is caused, most likely, by the detergent-like activity of *α*-TOS (Neuzil *et al*, 2002). That is, *α*-TOS comprises a hydrophobic phytyl chain (hydrophobic domain) and the chargeable succinyl group (functional domain), separated by the bulky tocopheryl moiety (signalling domain) (Neuzil, 2002). It is possible that *α*-TOS-induced apoptosis also involves lysosomal destabilisation by the agent, an action running parallel to and/or amplifying mitochondrial disruption (Neuzil *et al*, 2002). *α*-TOS may signal along several pathways. It has been shown that, due to its *α*-tocopheryl moiety, the agent activates protein phosphatase-2A that, in turn, inhibits protein kinase C (PKC). This leads to hypophosphorylation of the antiapoptotic protein bcl-2, with ensuing mitochondrial labilisation (Neuzil *et al*, 2001c). This pathway itself does not cause apoptosis by the VE analogues, but rather amplifies the succinyl moiety-mediated destabilisation of mitochondria, since *β*-, *γ*- and *δ*-TOS lacking the PKC inhibitory activity are less apoptogenic than the *α*-analogue (Neuzil *et al*, 2001c; Birringer *et al*, 2003). Other pathways that are likely implicated in apoptosis induced by *α*-TOS include deregulation of the c-jun/AP-1 (Qian *et al*, 1997) and the TGF-*β* pathway (Turley *et al*, 1995), as well as inhibition of the cell cycle transition (Turley *et al*, 1997a,1997b).

**Figure 1:**
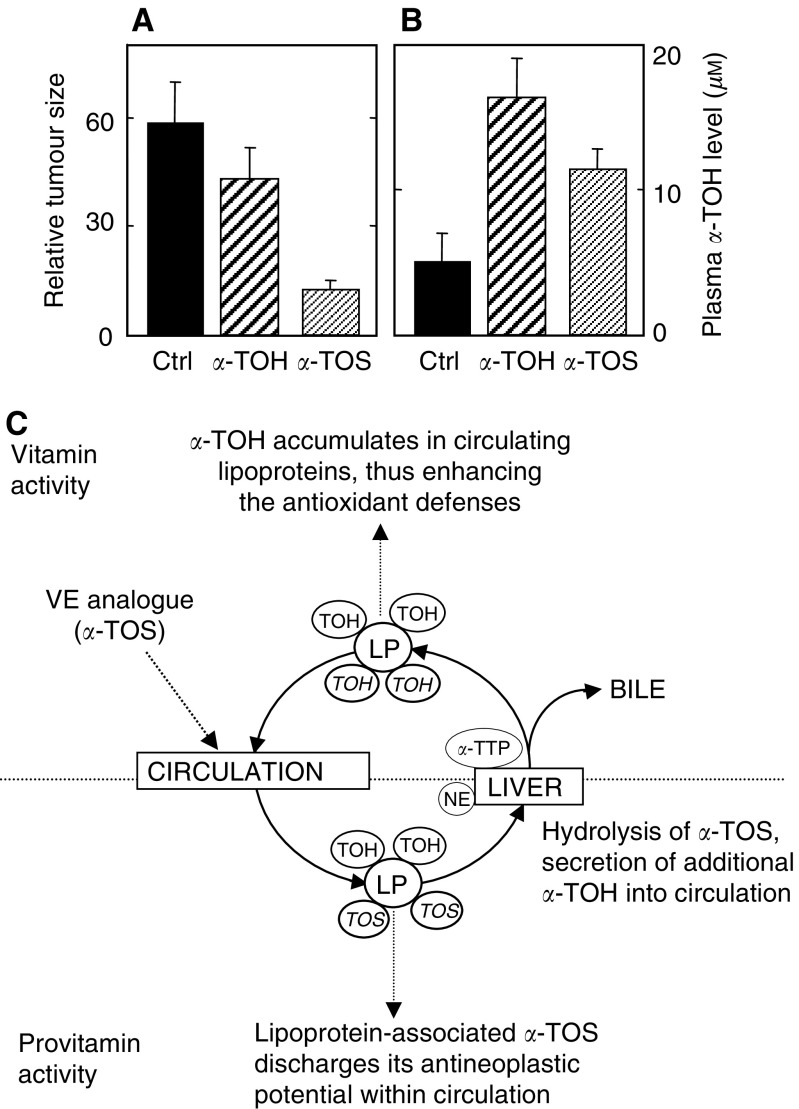
*α*-TOS inhibits colon cancer (**A**), and is converted to the redox-active *α*-TOH (**B**) (adapted from Weber *et al*, 2002). Nude mice with colon cancer xenografts were treated with the vehicle (control), *α*-TOH or *α*-TOS, by intraperitoneal injection of 100 nmol of the drug, every second day for 10 days. In the end, the tumour size was estimated (**A**) and the mouse plasma was analysed for the level of *α*-TOH (**B**). (**C**) Bases of the hypothesis are presented in this communication. After reaching the circulation, a VE analogue (epitomised in our studies by *α*-TOS) binds to circulating lipoproteins (LP), which carry it to the microvasculature, where it kills cancer cells (this is the ‘provitamin E effect’). Lipoproteins with bound *α*-TOS are cleared in the liver, where nonspecific esterase (NE) very efficiently cleaves it into *α*-TOH. The newly generated *α*-TOH is in part excreted in the bile, in part bound to the saturable *α*-tocopheryl-transfer protein (*α*-TTP), which inserts it into nascent very low-density lipoprotein. In this way, the provitamin E is converted to VE, which increases the antioxidant defences and acts as an immunosuppressive agent.

The strong apoptogenic activity of *α*-TOS translates to its anticancer efficacy, as shown in preclinical models. Malafa and co-workers have demonstrated the inhibition of breast cancer in a mouse model (Malafa and Neitzel, 2000), as well as suppression of melanomas (Malafa *et al*, 2002a) and colon cancer metastasis (Barnett *et al*, 2002). We have shown that the VE analogue suppressed colon cancer in an athymic mouse model, and its antineoplastic effect was much higher than that of *α*-TOH, which exerted only minor, nonsignificant cancer suppression (Weber *et al*, 2002). The great superiority of *α*-TOS over *α*-TOH in cancer inhibition is due to the fact that, while both agents inhibit proliferation of malignant cells in the xenografts, only *α*-TOS causes their apoptosis (Weber *et al*, 2002) (Figure 1A

). Another possible mode of antineoplastic activity of VE succinate is that it appears to be antiangiogenic (Malafa *et al*, 2002b). This notion follows also from findings that *α*-TOS causes apoptosis of proliferating, but not growth-arrested endothelial cells (Neuzil *et al*, 2002), and inhibits the expression of angiogenic cytokines, such as VEGF (Malafa and Neitzel, 2000). We have recently observed suppression of blood vessel sprouting by *α*-TOS in a mouse model of neonatal retinal neovascularisation (Neuzil *et al*, unpublished).

## PROAPOPTOTIC/ANTINEOPLASTIC ACTIVITY OF VE ANALOGUES IS STRUCTURE-DEPENDENT

The reason why *α*-TOS is proapoptotic while *α*-TOH does not possess this activity (in fact, *α*-TOH is antiapoptotic) has been investigated in considerable detail over the last few years. Importantly, Fariss *et al* (1994) showed that the cell-killing activity of *α*-TOS requires an intact molecule of the agent. They reported that the ester, which can be hydrolysed by some cells to free VE, has identical activity as its ether counterpart that is not hydrolysable. We have explored in more detail the structure–function relationship of these agents and found several interesting features (Birringer *et al*, 2003). Firstly, the presence of the phytyl group was essential for the activity, since the succinyl ester of Trolox was inactive. This can be explained by the fact that such a molecule is highly water soluble, therefore does not associate with lipid membranes. As mentioned earlier, lowering the number of methyl substituents on the tocopheryl moiety decreased the proapoptotic efficacy. Replacement of the succinyl group by an uncharged group, for example acetate, or its methylation, obliterated the proapoptotic activity. On the other hand, esterification of *α*-TOH with other dicarboxylic acids modulated the apoptogenic activity as follows: esters with higher number of carbons had lower activity than *α*-TOS, while esterification with unsaturated dicarboxylic acids, such as maleyl acid, highly enhanced the activity. Thus, *α*-tocopheryl maleate (*α*-TOM) is the most apoptogenic VE analogue tested to date (Birringer *et al*, 2003).

The unique structural features of agents like *α*-TOS probably make them selective for malignant cells (Neuzil *et al*, 2001b). While speculative at present, it is possible that the surprisingly high toxicity of *α*-TOS towards cancer cells and tissues is due to their persistence in such cells in the original form, while normal cells, including fibroblasts (Roberg *et al*, 1999), cardiac myocytes (Roberg and Ollinger, 1998), hepatocytes (Tirmenstein *et al*, 1999) and intestinal epithelial cells (Borel *et al*, 2001), are all capable of its hydrolysis to *α*-TOH. On the other hand, malignant cells, such as the human T lymphoma, malignant mesothelioma and colon cancer cells exert only a very low hydrolytic capacity towards *α*-TOS (Neuzil *et al*, unpublished). Another reason for the selectivity of *α*-TOS may result from its physicochemical properties. The VE analogue is a weak acid with a low p*K*_a_ value. Therefore, at the neutral pH of normal tissue interstitium, majority of *α*-TOS exists in the charged, deprotonated state. Since there are no known transporters of *α*-TOS, it can be expected that it will cross the plasma membrane at a low rate. On the other hand, the acidic pH of the tumour interstitium causes protonation of a substantial portion of the total *α*-TOS, facilitating its free diffusion into the cell. In favour of this hypothesis, lowering the pH of the cell culture medium enhanced the killing of cancer cells by *α*-TOS, but not by *γ*-tocotrienol, an apoptogenic VE analogue that cannot be deprotonated (Neuzil *et al*, 2002). This is consistent with the idea that inducers of apoptosis that are weak acids may be selective anticancer agents due to their preferential uptake by tumour tissue cells (Gerweck *et al*, 1999; Kozin *et al*, 2001).

It is therefore possible to modify the structure of VE analogues so that agents with higher proapoptotic activity and selectivity for malignant cells are generated. Thus, this group of compounds provides a unique opportunity for further structure–activity studies.

## VE ANALOGUES AS ADJUVANTS FOR CANCER IMMUNOTHERAPY

A commonly used practice in tumour treatment is to combine several anticancer agents with different modes of action. The reasons for this approach are both maximising the apoptotic potential of the cell, overcoming resistance to one of the agents by utilising complementing proapoptotic pathways and lowering the doses of the individual drugs to suppress the potential secondary deleterious effects of the treatment. From this point of view, *α*-TOS has shown substantial promise.

There are several reports documenting that the VE analogue potentiates cancer cells killing by the immunological apoptogen Fas, viz by mobilising the Fas receptor from the cytosol to the plasma membrane; this has been shown for both prostate and breast cancer cells (Turley *et al*, 1997; Yu *et al*, 1999). These findings suggest that *α*-TOS has the propensity to boost the immune system to enhance cancer surveillance. While the Fas ligand itself is highly cytotoxic, the TNF-related apoptosis-inducing ligand (TRAIL) is selective for cancer cells (Bonavida *et al*, 1999). Therefore, the reports that *α*-TOS can potentiate cancer cells to killing by TRAIL may be of pharmacological importance. This has been shown for both colon cancer (Weber *et al*, 2002) and mesothelioma cells (Neuzil *et al*, unpublished) as well as for T lymphoma cells (Dalen and Neuzil, 2003). One mechanism by which *α*-TOS sensitises cells to killing by TRAIL is that the two agents use different pathways of apoptotic signalling, that is, *α*-TOS activates the distant, mitochondrial pathway, while TRAIL acts through the proximal, receptor-mediated route (Weber *et al*, 2002). Moreover, the *in vitro* cooperative killing of colon cancer cells by the two agents has also been reported for the suppression of colon cancer in a preclinical model (Weber *et al*, 2002). Another possible mechanism of synergism and/or cooperation between TRAIL and *α*-TOS is the inhibition of activation of the nuclear factor-*κ*B (NF*κ*B). This transcriptional factor controls the expression of a number of prosurvival genes, and is activated in some cancer cells due to TRAIL crosslinking of one or more of its cognate receptors (Degli-Esposti *et al*, 1997; Bernard *et al*, 2001). *α*-TOS has been shown to suppress the activation of NF*κ*B (Suzuki and Packer, 1993; Erl *et al*, 1997), and we have shown that this may be due to the induction of proapoptotic signalling leading to caspase-3 activation that, in turn, cleaves the NF*κ*B subunit p65 (Neuzil *et al*, 2001a). A recent finding revealing that the suppression of NFκB activation by *α*-TOS, following exposure to TRAIL, sensitises T lymphoma cells to killing by the immunological apoptogen (Dalen and Neuzil, 2003), may be of pharmacological interest. Finally, *α*-TOS can sensitise cancer cells by upregulation of the TRAIL death receptors, as in the TRAIL-resistant mesothelioma cells (Neuzil *et al*, unpublished).

## THE PROMISE OF CANCER THERAPY BY VE ANALOGUES IS BASED ON PRECLINICAL STUDIES

Thus far, there have been very few studies on the anticancer effects of VE analogues *in vivo*. This is surprising, since the toxic effects of *α*-TOS towards malignant cells have been known since the early 1980s (Prasad and Edwards-Prasad, 1982; Prasad *et al*, 2003). Probably, the strongest evidence suggesting the potential use of VE analogues in the treatment of humans with neoplastic disease comes from the laboratory of Malafa. They first showed that *α*-TOS promoted beast cancer dormancy in an immunocompromised mouse (Malafa and Neitzel, 2000) and, later on, also melanoma dormancy (Malafa *et al*, 2002b); in both cases, inhibition of angiogenesis via suppression of VEGF signalling by *α*-TOS was suggested as the underlying mechanism, pointing to an indirect effect of the VE analogue on cancer growth. The acute effect of *α*-TOS has been shown by the same group for experimental melanoma (Malafa *et al*, 2002a) and colon cancer liver metastases (Barnett *et al*, 2002), and similar suppression of experimental melanomas has been observed by others (Kogure *et al*, 2003). In these experiments, the main mode of action of *α*-TOS was ascribed to its apoptogenic effect on the cancer cells. We have reported a significant inhibition of colon cancer growth in immunocompromised mice treated with *α*-TOS and its superiority to *α*-TOH (Neuzil *et al*, 2001c; Weber *et al*, 2002), and have also shown its cooperativity *in vivo* with the immunological apoptogen TRAIL (Weber *et al*, 2002). Finally, our recent studies revealed that *α*-TOS strongly enhances the survival of immunocompromised mice with experimental peritoneal mesothelioma, a thus far incurable type of human neoplastic disease (Neuzil *et al*, unpublished data).

Although these studies show a strong promise, there are certain drawbacks that need to be solved before *α*-TOS and similar compounds its possible application to humans. These compounds are esters of VE, and as such, are fully hydrolysed during the intestinal uptake following oral administration. Therefore, intravenous application is required. This can be overcome by generating analogues where the ester bond is replaced by an ether bond (Fariss *et al*, 1994), since ethers are not subject to enzymatic hydrolysis. An ether VE analogue has been recently reported as being highly effective in suppressing mammary tumours and lung metastases in syngeneic mice (Lawson *et al*, 2003). While this approach makes it possible to administer VE analogues orally, it jeopardises the most intriguing feature of the analogues based on VE esters in that the ether compounds are not broken down to the free VE (see below). Notwithstanding, the ether analogues of VE make it possible to administer the drugs orally, which has a clear advantage over intravenous application of the ester compounds, and we and others are pursuing this option as well.

## PHARMACOKINETICS OF VE ESTERS – A NOVEL PROVITAMIN-TO-VITAMIN CONVERSION MODEL

Perhaps, the most exciting aspect of the potential use of compounds like *α*-TOS, a pro-VE, follows from its pharmacokinetics. After infusion into the circulatory system, the VE analogue associates with circulating lipoproteins (Kayden and Traber, 1993; Pussinen *et al*, 2000). This carrier delivers it to the microvasculature of the tumour. Since there is a constant exchange of hydrophobic molecules between lipoproteins and the peripheral tissue, *α*-TOS can traverse to malignant cells, where it induces apoptosis (Neuzil, 2002). Due to rapid turnover of lipoproteins (Traber *et al*, 1994), *α*-TOS is eventually cleared in the liver. Hepatocytes have a high activity of nonspecific esterases that cleave the VE ester to *α*-TOH. Further, during processing of lipoproteins in the liver cells, the natural stereoisomer of *α*-TOH associates with the highly specific *α*-TOH-binding protein, which inserts this most active form of VE into nascent very low-density lipoprotein that is, in turn, resecreted into circulation via the hepatic vein (Terasawa *et al*, 2000). In this way, following hydrolysis of *α*-TOS, the circulation is enriched with VE. Therefore, the pro-VE, *α*-TOS and similar compounds (Birringer *et al*, 2003) are converted to the redox-active VE with additional beneficial activity (Neuzil, 2002), including increased protection against oxidative stress and immunosuppressive activity (Li-Weber *et al*, 2002). Moreover, several reports showed that *α*-TOS protects cells like hepatocytes from secondary deleterious effects of toxic agents, including adriamycin (Dominguez-Rodriguez *et al*, 2001; Fariss *et al*, 2001).

Based on direct and circumstantial evidence and on theoretical considerations, a novel hypothesis is proposed explaining why VE is inefficient as an anticancer agent and why its analogues may be deemed as potent anticancer drugs (Figure 1B). According to this hypothesis, VE succinate and similar analogues (in general, esters of VE with a free carboxylic group) induce apoptosis selectively in cancer cells while VE itself is not apoptogenic. Due to multiple activities, including apoptosis induction and sensitisation of cancer cell killing by other agents, VE succinate and its analogues inhibit cancer growth. Following the anticancer activity discharge, the pro-VE is converted to the redox-active VE, part of which enters the circulation.

Therefore, it is imperative to test this hypothesis, as it is believed that VE analogues, epitomised by *α*-TOS, represent an exciting group of novel anticancer agents of potential high pharmacological importance.
